# Insight into the Prospects for Nanotechnology in Wheat Biofortification

**DOI:** 10.3390/biology10111123

**Published:** 2021-11-02

**Authors:** Mohd. Kamran Khan, Anamika Pandey, Mehmet Hamurcu, Sait Gezgin, Tabinda Athar, Vishnu D. Rajput, Om Prakash Gupta, Tatiana Minkina

**Affiliations:** 1 Department of Soil Science and Plant Nutrition, Faculty of Agriculture, Selcuk University, Konya 42079, Turkey; mhamurcu@selcuk.edu.tr (M.H.); sgezgin@selcuk.edu.tr (S.G.); 2 Faculty of Agriculture, Institute of Soil and Environmental Sciences, University of Agriculture Faisalabad, Faisalabad 38040, Pakistan; tabinda_athar@yahoo.com; 3 Academy of Biology and Biotechnology, Southern Federal University, 344006 Rostov-on-Don, Russia; rajput.vishnu@gmail.com (V.D.R.); minkina@sfedu.ru (T.M.); 4 ICAR-Indian Institute of Wheat and Barley Research, Karnal 132001, India; op.gupta@icar.gov.in

**Keywords:** biofortification, cadmium toxicity, iron fortification, nanofertilizers, *Triticum aestivum* sp., zinc fortification

## Abstract

**Simple Summary:**

Wheat is a major crop consumed by a large population of the world. Hence, increasing its nutritional value can largely handle the malnutrition issues of the growing population. In the past few decades, different biofortification techniques including conventional breeding, transgenic approach, and agronomic biofortification have been largely employed for increasing the nutrient content in wheat grains. However, all of these techniques have their own drawbacks such as environmental hazards, long time requirement, reduced acceptability etc. Thus, nanobiofortification of wheat crop has gained interest as an efficient alternative strategy to achieve nutritional gains. However, there is still a long way forward to effectively utilize nanotechnology for wheat nutritional development. In this scenario, a review on the current advancement in wheat nanobiofortification is highly required so that the lacking points in this research area can be identified and accomplished. However, such a review article has been missing so far. This review describes the progress in the use of nanomaterials for wheat biofortification till date. It will help the scientific community to identify the lack in this research area and widely implement the nanotechnology to biofortify wheat crops.

**Abstract:**

The deficiency of nutrients in food crops is a major issue affecting the health of human beings, mainly in underdeveloped areas. Despite the development in the methods of food fortification, several barriers such as lack of proper regulations and smaller public-private partnerships hinder its successful implementation in society. Consequently, genetic and agronomic biofortification has been suggested as the potential techniques for fortifying the nutrients in diets. However, the time-consuming nature and restricted available diversity in the targeted crop gene pool limit the benefits of genetic biofortification. In agronomic biofortification, organic fertilizers face the problem of prolonged duration of nutrients release and lesser content of minerals; while in inorganic fertilizers, the large-sized fertilizers (greater than 100 nm) suffer from volatilization and leaching losses. The application of nanotechnology in agriculture holds enormous potential to cope with these challenges. The utility of nanomaterials for wheat biofortification gains its importance by supplying the appropriate dose of fertilizer at the appropriate time diminishing the environmental concerns and smoothening the process of nutrient uptake and absorption. Wheat is a major crop whose nano-biofortification can largely handle the issue of malnutrition and nutrients deficiency in human beings. Though several research experiments have been conducted at small levels to see the effects of nano-biofortification on wheat plants, a review article providing an overview of such studies and summarizing the benefits and outcomes of wheat nano-biofortification is still lacking. Although a number of review articles are available on the role of nanotechnology in wheat crop, these are mostly focused on the role of nanoparticles in alleviating biotic and abiotic stress conditions in wheat. None of them focused on the prospects of nanotechnology for wheat biofortification. Hence, in this review for the first time, the current advancement in the employment of different nanotechnology-based approaches for wheat biofortification has been outlined. Different strategies including the supply of nano-based macro- and micronutrients that have shown promising results for wheat improvement have been discussed in detail. Understanding several aspects related to the safe usage of nanomaterials and their future perspectives may enhance their successful utilization in terms of economy and fulfillment of nutritional requirements following wheat nano-biofortification.

## 1. Introduction

Food and Agriculture Organization of the United Nations revealed a prevalence of 9.9 percent undernourishment projecting that more than 700 million people faced hunger in the world in 2020 [1]. Additionally, more than 2 billion people are influenced by another form of malnutrition caused by the insufficient intake of micronutrients and vitamins and frequently known as hidden hunger [2,3]. A main reason behind this prevalence of malnutrition is the focus of our agricultural strategies toward increasing the crop production quantity and not quality. This led to a dearth of crucial nutrients in grain crops and consequently, among their consumers. Although the consumers have modified their food patterns and added nutritional food items such as dairy products, vegetables, fruits, and legumes to their diets, still a major population suffers from nutritional deficiencies [4]. This emphasizes the need of a nutritional revolution to enhance the nutritional value of crops [4,5]. Thus, agriculturists have diverted their focus toward employing several techniques to develop nutrient-rich crops.

Biofortification or “biological fortification” is one of such techniques that allow the growing of crops rich in nutrients at comparatively lower cost with increased accessibility to the human population. It also increases variability in the present nutrient content in the crops. Basically, two main types of biofortification, genetic and agronomic biofortification are being applied to increase the nutritional quality of crop products [6,7]. Both these methods offer an inexpensive strategy of supplying nutritious food to poor or undernourished people [8] and provide a sustainable, enduring and economical approach to deal with the issue of malnutrition. However, both these methods have their own limitations.

**Figure 1:**
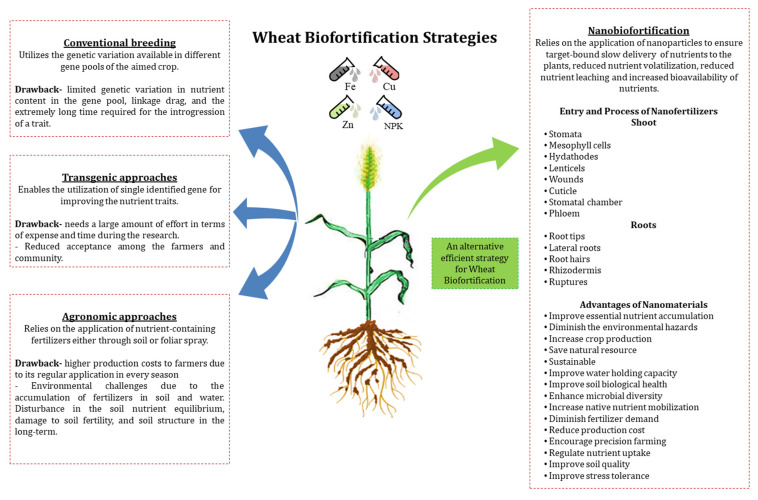
Different strategies used for wheat biofortification. The left side of the figure explains the wheat biofortification strategies other than nanobiofortification and their drawbacks. The right side of the figure provides a summary of nanobiofortification including its target, entry points of nanofertilizers in plants, and the advantages of nanomaterials (Fe—iron, Zn—zinc, Cu—Copper, NPK—nitrogen, phosphorus and potassium) [14,24,25].

Genetic biofortification involves both transgenic and conventional breeding approaches (Figure 1). Transgenic approach allows the transfer and expression of nutrient-based genes across different species fortifying even the crops with least nutritional value [9], and also reduces the anti-nutrients activity that regulates the bioavailability of nutrients in plants [2]. However, despite its positive aspects, transgenic approach needs a large amount of effort in terms of expense and time during the research [10]. Moreover, the reduced acceptance of transgenic crops among the farmers and community and difficult regulatory strategies for their commercialization also limit its benefits for the human population.

Similarly, biofortification via conventional breeding is the most acquired form of genetic biofortification due to its sustainable nature and great acceptability. The genotypes with higher nutrient value are crossed with the ones with comparatively less nutrient-rich but high-yielding genotypes [4] (Figure 1). A large number of projects and programs such as HarvestPlus and Health grain Project have largely invested in the biofortification of different crops including whole grain cereals such as maize and wheat, sweet potato (*Ipomoea batatas*), beans, pearl millet (*Pennisetum glaucum*) through conventional breeding. However, despite the potential advantages of conventional breeding programs, sometimes it is extremely difficult to employ them in biofortification programs due to limited genetic variation in nutrient content in the gene pool, linkage drag, and the extremely long time required for the introgression of a trait in commercial cultivars [11].

These limitations of genetic biofortification approaches suggest the implementation of agronomic strategies in biofortification programs. Agronomic biofortification mainly relies on the application of nutrient-containing fertilizers either through soil or foliar spray [7]. The application of macronutrients like nitrogen (N), phosphorus (P), and potassium (K) and micronutrients such as copper (Cu), zinc (Zn), iron (Fe), manganese (Mn) via agronomic biofortification provided higher yields and protected a major human population from starvation [12].

However, although agronomic biofortification is the most straightforward method among all the biofortification strategies, its success is dependent on several factors such as the type of nutrient to be supplied, differences in its uptake, movement, and accumulation across the plant tissues, and the property of the soil where the crop is grown [2]. Thus, there is a dire need of alternative and advanced strategies to fortify grown crops.

Nanotechnology is one of such emerging technologies that can be progressively applied in agriculture to fortify the crops and that can largely deal with the drawbacks of genetic and traditional agronomic biofortification [13]. Nanomaterials especially in the form of nanofertilizers (NFs) have the capacity to mitigate different stress conditions and enhance crop yield by increasing the nitrogen metabolism, seedling growth, carbohydrate and protein synthesis, photosynthesis, and increased translocation of nutrients from roots to leaves [14]. Moreover, comparatively, a smaller amount of nanofertilizer application can effectively enhance the nutrient content with lesser harm to the surrounding environment due to its target-bound slow delivery [15].

Wheat being the major staple crop has always been the center of attraction for different types of biofortification [4]. Similar to other biofortification strategies, a number of studies revealing the positive outcomes of nanomaterials on wheat nutritional content have been conducted in greenhouses or small-scale field experiments to date [16,17,18]. However, nanotechnology could not be effectively employed for wheat biofortification in large-scale agricultural programs.

To increase the employment of NFs in large scale wheat biofortification programs, it is necessary to have an overview of the effect of different nanomaterials and their application strategies on the nutrient content of the wheat genotypes [19]. However, a review summarizing the utilization of nanomaterials for wheat biofortification is lacking till date. Hence, to fulfill this lack, in this review, the advancement in the employment of nanomaterials to fortify wheat seeds/crops with different nutrients until date has been deliberated. Moreover, the opportunities and benefits lying in the utilization of different nanotechnology-based methods for wheat biofortification have been discussed. Besides the challenges, nanobiofortified wheat crops hold a bright future to address the malnutrition challenge. The studies that have been discussed in this article can be taken into account while planning the application of different NFs in the wheat fields for the nutrient biofortification.

## 2. Advantages of Nanobiofortification over Agronomic Biofortification

Cereal grains including wheat, maize, and rice are the major food crops supplying around 60% of the daily calorie requirements in developing countries [20]. Though these are rich in nutrient sources such as carbohydrates, fibers, proteins, lipids, vitamins, minerals, and phytochemicals as whole grain products, most of their nutritional components especially micronutrients are lost due to conventional processing [21]. Consequently, fortification of wheat, rice, and maize flour with different vitamins and minerals especially iron and zinc is being emphasized in several countries that further enter in the food chain via flour-based food products [22]. However, due to lack of sustainability in supplement fortification methods, other innovative strategies are required to ensure sustainable micronutrient levels in grain crops.

Agronomic biofortification has been considered as one of the efficient techniques increasing the mineral content in cereal crops at large scale. However, agronomic biofortification is not efficient to meet the expectations either due to poor quality of soil or due to less-efficient drainage system, and consequently, most of the applied fertilizers are wasted [23]. A loss of around 40–70%, 80–90%, and 50–90% of applied N, P, and K fertilizers respectively have been reported previously [14]. The requirement of their regular application in every season enhances the production costs to the farmers. Their repeated applications decrease the soil fertility and enhance salt content in soil leading to future losses in crop yields. The uncontrolled nutrient release via chemical fertilizers worsens the quality of the crop grains. Other than these issues, as the chemical fertilizers are being applied in greater amounts, the larger accumulation of toxic by-components including nitrate, heavy metals etc., in soil and water is one of the biggest challenges that agronomic biofortification poses on the environment. Accordingly, the disturbance in soil nutrient equilibrium, damage to soil fertility, and soil structure are the long-term consequences of the application of chemical fertilizers [24] (Figure 1). Consequently, there is a crucial need to find an efficient strategy to enhance quantity and quality of crops without increasing the annual consumption of chemical fertilizers.

Nanotechnology could provide possible solutions to several drawbacks of agronomic biofortification. Several properties of nanomaterials such as high sorption capacity, slow and controlled release at target sites, and high surface to volume ratio make them appropriate for nanofertilizers production [25]. The encapsulation of nutrients with nanomaterials leads to efficient absorption of nutrients by plants due to slow or control release of nanoparticles, and easy penetration of biological barriers by nanoparticles entering the plant vascular system [14,26,27]. This steady long-term delivery of plants via nanofertilizers allows enhanced crop growth as compared to conventional fertilizers [25]. As nanofertilizers are added in small amounts, these also prevent soil to get burdened with the by-products of chemical fertilizers [28] and reduces the environmental hazards. Contrary to chemical fertilizers, nanofertilizers can be synthesized and supplied according to the nutritional demand of the crop and status of the soil nutrients using biosensors [29]. Additionally, as compared to chemical fertilizers, nanofertilizers enable high bioavailability of minerals to the plants due to their smaller size, high reactivity, and higher surface area [30]. Thus, due to these mentioned advantages of nanofertilizers over the chemical fertilizers, these have been constantly being preferred for wheat biofortification nowadays.

## 3. The “Nano” Forms Used in Biofortification Programs and Their Types

NFs are widely used for the controlled release of nutrients into the soil that can eventually uplift the availability of nutrients to different plant organs leading to the improvement in its yield and quality [31]. Due to their ability to cover a wider surface area and their efficient absorption by plants, NFs are more supportive toward plant development and environmental safety as compared to the parallel amount of conventional fertilizer (Figure 1). These are applied in lesser quantities causing diminished leaching and reduced gas emissions to the atmosphere [32,33,34]. The efficiency of NFs varies according to their composition, size, chemical features, and especially the crop for which it is used [13]. NFs are elucidated as the compositions of very small size usually equal to or less than a nanometer in size comprising of macro and microelements including N, P, K, magnesium (Mg), calcium (Ca), sulfur (S), Fe, Zn, Cu, Mn, boron (B), nickel (Ni), molybdenum (Mo) and their compounds such as cerium oxide (CeO_2_), titanium oxide (TiO_2_), silver (Ag), gold, zinc oxide (ZnO), iron oxide (FeO), carbon nanotubes, aluminum oxide Al_2_O_3_ etc. [24,35,36,37].

Three different types of NFs are being successfully used for biofortification programs: (i) Nanoscale coating fertilizers where conventional fertilizers are encapsulated by nanoparticles (NPs) or intercalated in nanopores (such as zeolites and nanoclays) either to help the delivery or delay the release of a nutrient or to supplement with an additional element at a nano-level [38,39,40,41]; (ii) nanoscale additives fertilizers where conventional fertilizers are supplemented with NPs of a nutrient; (iii) nanoscale fertilizers or NFs are the NPs containing nutrients themselves that are directly used as fertilizer and each particle is less than 100 nm in size [37].

Nanomaterials that are utilized for wheat biofortification can also be classified as (a) Polymeric nanomaterials made up of repeated chains of molecules differing in structure and compositions [42]; (b) ceramic nanomaterials that are nonmetallic and heat resistant nanomaterials composed of both metallic and nanometallic compounds [43,44]; and (c) metal nanomaterials made up of metallic compounds such as metal oxides, quantum dots, nanogold, nanosilver [45]. Moreover, these materials can be grouped according to their nutritional benefit to the applied plants, such as (a) micronutrient fertilizers, (b) macronutrient fertilizers, (c) plant growth-stimulating NFs, and (d) nanomaterial-enhanced fertilizers [44].

In the next sections, the successful employment of these fertilizers for wheat biofortification has been discussed in detail.

## 4. Micronutrients Nanobiofortification in Wheat

Micronutrient NFs consist of micronutrients such as Zn, Fe, B, Mn, Cu etc., that are required by plants in smaller amounts as compared to the macronutrients [14]. These are mostly added to the composite NPK fertilizers in small quantities. However, the availability of micronutrients to the plants can be low causing their deficiency and reduced plant growth. Micronutrient NFs due to their small size and increased surface area can increase the bioavailability of these micronutrients, also facilitating their accumulation in sink organs [30]. Among all the micronutrients, NFs of Fe and Zn are mostly focused due to the prevalence of their deficiency in the world population. Consequently, like other crops, in wheat also, the effect of Zn and Fe NFs on growth and development has been mostly studied.

Zn deficiency has devastating effects on wheat growth causing necrosis in leaf tissues, reduction in plant growth, decreased seed quality, and consequently, affected yield [46]. It also has considerable effects on humans leading to growth and health disorders. Around 50% of global land suffers from Zn deficiency. Though the demanding cultivation of cereal crops in highly alkaline soils rich in calcium or phosphorus and poor in organic carbon mainly accounts for the reduced Zn availability, the formation of insoluble compounds such as zinc carbonate or hopeite and its binding with compounds such as iron oxyhydroxides also reduces the Zn bioavailability [47]. Even the water-soluble Zn fertilizers are not completely available for plants due to their precipitation after interface with phosphates, carbonates, and calcium in the soil. Thus, biofortification is the immense need of time to overcome the Zn deficiency both in plants and humans. Similar to Zn deficiency, Fe deficiency is largely being discussed as one of the most prevalent reasons for malnutrition in the world causing anemia. The recommended daily Fe intake can be provided by biofortifying the staple crops such as wheat with Fe. Although genetic and agronomic biofortification methods have been successfully used in several programs for increasing wheat Fe and Zn content [4], the employment of nanobiofortification for enhancing wheat Zn and Fe has been limited. However, there are a few potential studies that have effectively implemented nanobiofortification for improving wheat growth and quality along with the Fe and Zn levels.

## 5. Macronutrients Biofortification in Wheat

Macronutrient NFs are composed of one or more macronutrients (such as Ca, Mg, K, N, and P) encapsulated or intercalated with nanomaterials. As plants need more amount of macronutrients especially NPK for their growth and production, it is assumed that global utilization of composite NPK fertilizer will increase up to 263 million ton by 2050 [48]. Though this employment of NPK fertilizers will largely contribute to per-capita wheat production, it will also serve as a source of soil and water pollution [49]. In this scenario, the enhanced use of macronutrient NFs in wheat production can be a more environment-friendly and sustainable approach. Understanding this urgent need, several studies have been conducted to observe the effect of macronutrient NFs on wheat yield and quality.

## 6. Entry of Nanoparticles into Wheat Plants and Their Effect on Nutritional Composition

Method of application of nanoparticles has a great effect on the extent of accumulation of a particular nutrient in wheat plants. However, the reason behind this is not completely understood. Thus, studies should be conducted to understand the mechanism of uptake, absorption, and translocation of NPs in wheat plants via different application methods. During the uptake and translocation, NPs pass through different root and shoot tissues which have specific size exclusion limits (SELs) and thus, act as barriers [50]. For example, cell walls with SEL of 5–20 nm restrict the movement of NPs in apoplast. Nevertheless, it has also been reported that some NPs of size 36–50 nm can enter and translocate by making structural changes and inducing the formation of larger and new pores in different tissues [50,51].

Among the soil and foliar application, foliar application is considered to be a comparatively more efficient way of nutrient delivery in plants as the soil properties may hinder the plant bioavailability of nutrients. However, foliar agronomic biofortification via conventional formulations can lead to leaf tissue injury due to the fast release of high amounts of ions into leaf tissue that can be locally phytotoxic. In that case, foliar application of NPs offer a sustainable strategy by providing a slow controlled release of ions into leaf tissue and avoiding the necessity for multiple applications. The foliar application of Fe and Zn NPs showed greater increase in root-shoot lengths, leaf area, dry weights, yield and photosynthesis rate as compared to soil amendment [52,53].

In foliar application, cuticle pores and stomata are considered as the two main pathways for the entry of NPs. However, as the size of the cuticle pores range from 0.2 to 2 nm, a number of NPs cannot be absorbed via these pores [50]. Zhu et al. [54] reported stomata as the main route of entry of ZnO NPs into wheat leaves on foliar application. The ZnO NPs accumulates under stomata on entry and after dissolution in the apoplast, Zn ions get distributed in plant leaves via entering into mesophyll cells [55]. Other than leaves, the foliar exposure of ZnO NPs are found to have increasing Zn content in various parts of wheat plants depending on the concentration of the applied NPs. A foliar treatment of 100 mg/L of ZnO NPs showed Zn concentration of 100–150 mg/kg DW in root-shoot tissues, and 45 mg/kg DW in grains of wheat plants [56]. A soil application of 100 mg/kg ZnO NPs revealed similar concentration of Zn in root, shoot, and grains of wheat plants [57].

Though foliar ZnO NPs are found to be increasing the wheat grain zinc content, the increment is lesser as compared to conventional Zn formulations [58]. Foliar application of chitosan-complexed Zn NPs post anthesis showed grain Zn concentration of approximately 21–27 µg/g. Moreover, foliar application of ZnO NPs shows grain Zn accumulation in the crease region, aleurone layer and embryo similar to soil uptake [58]. The results of a foliar spray of 2 g/L ZnO NPs and 7g/L ZnSO_4_ on wheat plants grown in field showed that ZnO NPs not only increases the Zn concentration in the crease and aleurone, but also in the endosperm. Similar to ZnO, foliar application of Fe NPs also increases Fe concentrations in wheat tissues and grains in a dose additive manner. On the one hand, where the soil applications of Fe NPs showed higher shoot Fe concentrations than foliar supply, the grain Fe concentrations are found to be greater in foliar application (110 mg/kg) as compared to soil application (90 mg/kg) [53]. The soil application of Fe NPs have shown approximately 50, 90, and 47 mg/kg DW Fe concentration in shoot, root, and grains of wheat plants [59]. Similarly, wheat seeds priming with ZnO and Fe NPs before sowing not only increased the Fe-Zn concentration but also reduced the Cd concentration in roots, shoots, and grains.

The uptake of nutrients by wheat plants from NPs largely varies according to the soil type, its interaction with the applied NPs, and the type of application [60]. As mentioned briefly in this section, to date, three main methods including seed priming, foliar application, and soil application have been used for the application of nanomaterials to the wheat plants, and all of these methods have their advantages and effects. In the next sections, the employment of these methods for wheat nanobiofortification has been discussed in detail.

## 7. Wheat Micronutrients Nanobiofortification via Seed Priming

**Figure 2:**
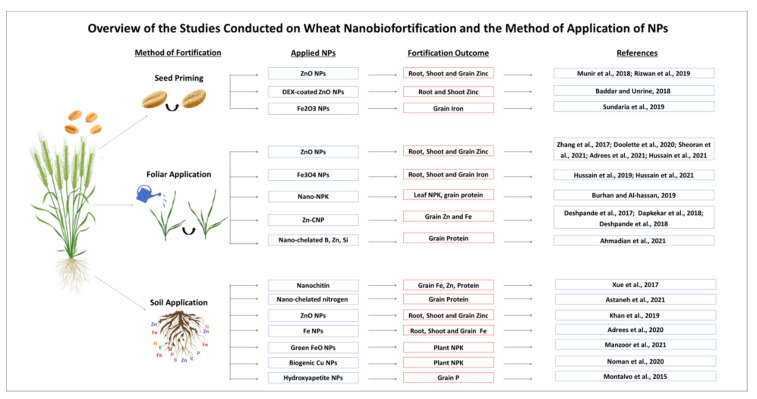
An overview of the different methods used for Wheat Nanobiofortification, types of applied nanomaterials, and their outcome in the form of fortified nutrients. (Abbreviations in the figure: NPs- nanoparticles; ZnO—zinc oxide; dex—dextran; Fe_2_O_3_—ferric oxide; Fe_3_O_4_—ferrosoferric oxide; NPK—nitrogen, phosphorus, potassium; CNP—chitosan nanoparticles; B—boron, Fe—iron, Zn—zinc; Si—silicon; P—phosphorus; Cu—copper).

Seed priming is a method that enhances the plant’s potential for nutrients uptake and translocation [52]. This method provides hydration to seeds in a regulated manner up to the germination stage but before the protrusion of radical. In seed priming, seeds are treated with different amendments prior to sowing so that those can proceed toward different biochemical and metabolic progressions required for germination [61]. This stimulates different physiological processes in plants to deal with devastating effects of abiotic stresses and enhancing the nutritive value of crops along with their yield [52,62,63]. Seed priming not only decreases the duration of seedling emergence but also allows uniform germination with enhanced germination rate [64,65,66,67]. The fortification of nutrient crops by seed priming via conventional chemicals and fertilizers has been in practice since last several decades. Seed priming with NPs can further improve seed metabolism, deliver nutrients more efficiently than conventional forms, and have the benefits that nanofertilizers possess over the conventional fertilizers [68]. Several studies have utilized NPs for the priming of wheat seeds to fortify the staple crop with nutrients (Table 1, Figure 2). Elhaj Baddar and Unrine [47] suggested that ZnO NPs can be effectively used to treat wheat seeds to enhance Zn nutrition. They investigated the effect of seed treatment with bare ZnO NPs, dextran (DEX)- and dextran sulfate (DEX (SO_4_))-coated ZnO NPs on wheat growth and Zn accumulation as compared to the conventional zinc sulphate (ZnSO_4_) fertilizer. All the NPs-based formulations used in their study showed increased Zn concentration in wheat seeds in comparison with ZnSO_4_, where bare ZnO and DEX-coated ZnO NPs showed maximum Zn concentration in shoot. The type of NP to be used for biofortification depends on the targeted objective as the surface chemistry influences the distribution of Zn within the plant and the growth of the tissue and biomass. The type of charges of the coating materials also affects the uptake, translocation, and accumulation of Zn in different wheat tissues [69]. Moreover, the growth responses may vary according to both the concentration and the type of Zn treatment [16]. The increase in the Zn concentration of shoots, roots, and grains of wheat plants can be proportionate [16] or disproportionate [17] to the increasing concentration of the applied ZnO NPs in priming of wheat seeds, though an increment is always observed. However, most of such studies have been conducted in hydroponics or pots and their efficacy in field experiments should be thoroughly determined. Seed priming with Zn and Fe NPs are not only seen to have a positive effect on the accumulation of these elements in wheat grains but is also known to have simultaneous suppressing effects on the toxicity of heavy metals such as cadmium (Cd), reducing their root, shoot, and grain accumulation [17]. The wheat grain Cd content can be decreased below the threshold level of 0.2 mg kg^−1^ when seeds are primed with higher Zn and Fe NPs. This decreased Cd concentration can be attributed to the simultaneous competition for uptake of Cd, Zn, and Fe at the root surface of wheat plants and consequently, improved uptake of Fe and Zn from the Fe_3_O_4_ and ZnO NPs [17]. Thus, seed priming with NPs can be considered as a promising method of wheat Zn and Fe nanobiofortification not only in the normal growth conditions but also in the Cd stressed growth conditions and can also solve the problem of reduced germination rate of wheat seeds up to a certain extent.

Seed priming of wheat seeds with different concentrations of FeO nanoparticles (IONPs) leads to the accumulation of different concentrations of Fe in wheat grains [52]. A 12 h soaking of wheat seeds of two contrasting genotypes, IITR26 and WL711, showed a noticeable increase in grain Fe content even at the 25 ppm supply of IONPs. The former genotype with significantly higher concentrations of Fe, Zn, and manganese showed around 46% increase, while the later low-iron genotype showed an increment of 27% in grain Fe content. Wheat seeds priming with Fe showed stimulation of Fe transport mechanism and consequently, an accumulation of Fe in different sections of the seeds including the aleurone layer, endosperm, nucellar projection, and pigment strand of the crease [52]. Thus, similar to Zn, for Fe biofortification also, wheat seed priming is an effective strategy for grain Fe acquisition and accumulation.

## 8. Wheat Micronutrients Nanobiofortification via Soil Fertilization

A number of studies revealed the increase in grain nutrient content on soil application of nanofertilizers as well (Figure 2, Table 1). The soil application of FeO nano fertilizers not only fortifies wheat grains with enhanced Fe content but also reduces the Cd accumulation under the combined Cd and drought-stressed wheat. The alleviation of oxidative stress developed by drought and Cd stress can be one of the major modes of action of IONPs. However, the success of this application also lays in the concentration of the NPS applied [59]. Similar to Fe, Khan, et al. [57] demonstrated the effect of soil application of ZnO NF on the growth of wheat plants in either only Cd-stressed soil or combined with water stress. While an increment in Cd content in wheat tissues was observed under drought stress, the Zn NF supply reduces the Cd uptake and accumulation by roots. An enhanced grain Zn concentration on ZnO NF application under Cd and drought-stressed environment emphasizes the utility of NF in wheat biofortification even in the contaminated soils. Moreover, it is observed that due to the slow release of nutrients, soil-applied ZnO NFs can stay in the soil and can be used by upcoming season’s crops.

Other than chemically synthesized NPs, biologically synthesized NPs which are popularly called “green NP” are gaining great attention nowadays due to their less toxic and environmental friendly nature along with their cost-effectiveness. Green FeO-NPs developed from bacterial strain *Pantoea ananatis* RNT4 when applied to wheat plants via soil application co-ameliorates the effect of salinity and cadmium stress. At the same time, it can also biofortify the wheat plants with macronutrients, N, P, and K showing an increase of 33, 35, and 33% respectively on 100 mg kg^−1^ supply of FeO-NPs as compared to no NPs treatment [70]. Similarly, biogenic copper NPs synthesized from a copper-resistant strain of bacteria *Shigella flexneri* SNT22 regulate the Cd stress by minimizing the movement of Cd from soil to plants and simultaneously increase the concentration of macronutrients such N, P, K, and Ca [71]. This reduction in Cd translocation can be attributed to the struggle between Cd and CuNPs for entry into the root epidermal cells. Moreover, similar to ZnO and Fe NPs [17], the restricted intake of Cd into wheat plants can be due to the use of the same transport channels by both Cu and Cd. Thus, the higher intake and consequently higher concentration of Cu in the plant restrict the Cd uptake [72].

Other than increasing the biomass and grain yield, ZnO NPs are found to be non-toxic to plants even at a higher dose of 200 ppm. A significant increase in grain Zn content but not in leaf Zn of wheat plants was observed in the application of ZnO NPs via soil when compared with the ZnSO_4_ fertilizers [73]. Dimkpa, et al. [74] determined the shared effect of soil application of ZnO nanoscale and bulk particles, organic fertilizer, and drought on the mineral accumulation of wheat grains. The bulk-ZnO and nano ZnO increase grain Zn content by 23 and 39% respectively as compared to control, while the addition of organic fertilizer can increase this content up to 94% under drought condition. This suggests that the addition of organic fertilizers along with the nano ZnO can be successfully applied in wheat biofortification programs especially in water-deficient growth environments. However, the reducing effect of both nano ZnO and bulk ZnO on wheat grain Fe content either in the presence or absence of organic fertilizer under drought conditions should be considered while programing a wheat biofortification program [74].

The soil application of nanochitin, which is obtained from the hydrolysis of shrimp chitin, has a positive effect on the growth rate and yield of wheat plants. An increase of 22.1, 10.3, and 5% in Zn, Fe, and protein content of multi spike wheat; and an increment of 27, 32, and 33.4% in Zn, Fe, and protein content of large spike wheat was respectively obtained by treating wheat plants with 6 mg kg^−1^ nanochitin [75].

Although a number of studies have been conducted to observe the effect of soil application of nanomaterials on the nutrient content of different wheat tissues, none of the studies (except Astaneh et al. [76]) have included conventional fertilizers in their experiments for comparison. The actual advantage of nanomaterials as compared to the conventional fertilizers for wheat biofortification can be properly understood by including both of them in the study designs.

## 9. Wheat Micronutrients Nanobiofortification via Foliar Fertilization

Foliar application of NFs has also been successfully employed for improving the quality of wheat grains under stressed and non-stressed conditions (Figure 2). The foliar application of nano-Fe_2_O_3_ fertilizers on wheat plants is found to be more effective than the same amount of iron chelate and iron sulfate in increasing the chlorophyll, grain protein, and grainFe content [77]. Similarly, the foliar application of Fe NPs was found to be more efficient than soil application in terms of Fe biofortification of wheat grains and reducing the grain Cd concentration under Cd-contaminated soil [53]. However, both methods are promising for Fe nanobiofortification in wheat grains. Additionally, foliar application of NFs gives the advantage of their supply with pesticide application in the field. In contrast to soil application, foliar application of NF ensures the decreased passage of NPs to soil compartments and water, thereby reducing the chances of environmental pollution.

Some of the studies aimed to observe the effect of foliar application of nano ZnO fertilizer on the growth of wheat plants revealed more than 20% increment in grain protein content and a significant increase in photosynthetic pigments as compared to the conventional Zn fertilizer foliar spray [78]. Along with increasing the wheat grain protein content, foliar application of nano ZnO also reduces the leaching of Zn into the soil after harvest [78]. Moreover, a lesser amount of NPs is required for grain Zn biofortification in the foliar application as compared to the soil application [18,57]. Similar to the seed priming method [52], the foliar application of ZnO NPs on wheat plants also lead to the accumulation of Zn in the crease and aleurone layer of the wheat grain along with a slight increment in endosperm Zn making it an appropriate method for Zn biofortification [79]. The Zn accumulated in leaves from foliar supply of ZnO NFs can be efficiently utilized in metabolic processes of plants [80,81]. This accumulation can be attributed to the direct absorption of ZnO NPs by the leaf cuticles of wheat plants and their movement across the leaf epidermis via stomata, their release in apoplast and then adhesion to the mesophyll cells irrespective of the particle coating [54,82].

Similar to soil application of Fe NPs [59], foliar application of ZnO NF also increases the plant growth, diminishes grain Cd, and enhances wheat grain Zn concentrations under Cd-stressed condition and under combined drought and Cd-stressed condition [18]. The decrement in Cd concentrations on ZnO NFs application can be due to the higher Zn concentrations in plants. The Zn transport can be suppressed by the presence of higher Zn in plants as Cd and Zn may use the same transporters. The foliar-applied ZnO NPs improve the chlorophyll content of the treated plants, enhances the Zn transport, lowers the Cd content, increases the biomass, and also enhances the translocation of Zn to grains [58]. Foliar application of both Fe and Zn NPs simultaneously on wheat plants grown in field conditions reduce the Cd uptake while enhances the Fe and Zn biofortification in wheat grains in Cd contaminated soils [56]. This serves as a method for simultaneous Fe and Zn nanobiofortification of wheat. This simultaneous Fe-Zn biofortification of wheat grains in Cd contaminated soil is also possible when Si NPs are supplied with Fe and ZnO NPs [56].

Chitosan (CHT) NPs which come under the category of nanomaterial-enhanced fertilizers or polymeric nanomaterials are actively being used for the delivery of agrochemicals in wheat [50]. CHT that is the deacetylated form of chitin is the second most significant biopolymer after cellulose on earth. The efficiency of foliar application of zinc complexed chitosan nanoparticles (Zn-CNP) as nano-micronutrient carrier for wheat biofortification has been observed by Deshpande, et al. [55]. Foliar ZnCNP not only enriches the durum wheat grains with 27 to 42% Zn in zinc-deficient growth conditions but also enhances its translocation to both leaf and seeds and inhibits the nutrient loss to the soil [55]. Moreover, ZnCNPs are known to improve the Zn utilization efficiency of wheat plants if applied at right time in the right doses at the right place and then can Zn fortify the wheat grains by 36% even in the low Zn supply of 40 mg/L [83]. This can be due to the effect of applied ZnCNP on the expression of the genes related to metal homeostasis, including the Fe and Zn-regulated transporter-like proteins that show a significant relationship with the grain Zn content [84].

Si is the second most abundant element in the earth’s crust, which in the form of fertilizer decreases the pH and enhances the nutrient absorption from the soil. The soil and foliar application of SiO_2_ NPs on wheat plants grown under drought stress conditions can increase wheat yield by 17.81% and 23.35% respectively [85] with a simultaneous increase in grain protein content. Ahmadian, et al. [86] observed the effect of foliar application of three different nano-chelated fertilizers comprising B, Zn, and Silicon (Si) on the growth rate and grain protein content of wheat plants grown under water-deficit conditions. The two-year experiment showed an increase in grain protein content by all the nano-fertilizers as compared to control either under fully irrigated or drought-stressed conditions. However, the increment was highest in nano-Zn treatment followed by nano-Si and it was higher in water-deficient condition than the fully irrigated regime. This increase might be due to the decrement in yield under drought conditions that leads to an increase in nitrogen content in grain [85]. Moreover, as Zn supply is known to be affecting the gene expression and protein synthesis in plants, this might be a reason for the increased protein content [87]. Similarly, Si fertilizer reduces the membrane damage in plants and thus, can be involved in increasing the protein content [88].

The studies on wheat nanobiortification employing foliar application include both pot-based and field-based experiments (Table 1). However, most of them were Fe and Zn-based nanobiofortification. More studies should be conducted to fortify other micronutrients in wheat via nanobiofortification.

## 10. Other Diverse Aspects of Wheat Nanobiofortification

Not only amount, but the size of applied NPs also affects the nutrient uptake. In a hydroponic experiment, Al-Amri, et al. [89] demonstrated that medium-sized (20–40 nm) iron (III) oxide (Fe_2_O_3_) nanomaterials are more efficient for enhanced Fe uptake, their translocation to wheat leaves, enhanced photosynthetic activity and consequently enhanced biomass as compared to the smaller (8–10 nm) and bigger (30–50 nm) IONPs.

Other than having beneficial effects, nano ZnO may have toxic or lethal effects due to their increased reactivity developing from their small size and larger surface area [90]. The effects of nano ZnO vary according to the plant species, growth stage, type and period of application, and the applied doses [91]. Thus, to minimize the extent of lethal effects, it is suggested to use ZnO NFs at appropriate doses. Moreover, although NFs reduce the quantity of applied nutrients thereby decreasing the input costs, their large-scale application does not seem realistic in the current scenario due to potential aggregation, dissolution, and large drift in the air. Thus, a competent strategy is required to deliver nano-scale nutrients to plants. The coating of bulk fertilizers with NPs can be an efficient method to handle the issues related to the dissolution and segregation of nano fertilizers [92]. In a study conducted by Dimkpa et al. [90], the effect of urea coated with ZnO-NPs on the growth of wheat plants grown in drought conditions as compared to non-coated urea and urea amended with ZnO-NPs was observed. Along with an increase of 51% in grain yield, nanoZnO-coated urea showed a significant increment of 24% in Zn uptake suggesting that the supply of urea coated with ZnO-NPs can increase the wheat growth and Zn accumulation under large-scale cultivation.

Wheat plants are not only exposed to freshly applied nano ZnO always but can also interact with the weathered ZnO NPs present in the soil. The weathered or aged ZnO NPs can have a different effect on the accumulation of Zn and other ions in wheat grains. In a study, where the effect of weathered nano ZnO on grain Zn content was observed, weathered nano Zn showed an increase of 186% in grain Zn content, while the fresh nano ZnO demonstrated an increment of 229% [74].

Other than micronutrients, the wheat nanobiofortification with macronutrients via soil application of NFs have been previously reported. The soil application of 41 kg ha^−1^ nano-chelated nitrogen fertilizer containing 17% nitrogen can increase up to 26% phosphorus, 6% potassium, and 52% protein in wheat plants even in drought-stressed conditions [76]. The bioavailability of P in plants, even in P-rich soils, is largely limited by the fixation of the P released from the soil organic matter in the form of insoluble inorganic compounds leading to P deficiency. Thus, the target is to develop fertilizers that can allow the slow release of P to increase the bioavailability at different growth stages. Hydroxyapatite nanoparticles (nHAP) are the form of macronutrient NFs that belong to the calcium phosphate compounds and are being employed as a source of slow-release of P and other nutrients in plants [93]. In this regard, a comparison of nHAP was done with the bulk HAP and triple superphosphate (TSP) for the P availability to *Triticum aestivum* plants [94]. In the experiment, although the phosphorus uptake in plants was less in nHAP than TSP, it was much better than bulk-HAP due to the restricted movement of bulk-HAP in soil. Thus, the utility of nHAP for biofortification of wheat with macronutrients cannot be ignored and should be thoroughly explored in wheat biofortification programs.

Similar to soil application, the foliar application of NFs also delivers the macronutrients and fortifies the wheat grains. The foliar application of liquid nano NPK fertilizers showed an increment of 19.37% in N, 44.11% in P, 12.03% in K, and 27.24% in protein content of wheat genotypes as compared to the traditional NPK fertilizers. However, different genotypes showed a different extent of nutrient accumulation due to variation in their growth rates [95].

Unfortunately, the advantages of nanomaterials for wheat biofortification with macronutrients have been explored to a very limited extent. Thus, more studies should be conducted in this direction especially for NPK biofortification so that the large application of conventional NPK fertilizers can be reduced and environmental damage can be reduced.

## 11. Wheat Nanobiofortification in the Light of Cost Effectiveness and Human Health

It has been discussed in different sections above that the employment of nanofertilizers for wheat biofortification reduces the production cost to the farmers. As NPs can be applied in appropriate doses at appropriate timings, these can optimize the use of natural resources allowing “precision farming”. While conventional fertilizers are being applied several times in a growth season by farmers to increase the yield, the controlled release of nutrients from nanofertilizers reduces the number of doses and consequently application costs [44]. A chemical fertilizer consumption of 22 million tons have been reported in 2015 by USDA [96]. Most of this applied fertilizer is wasted due to leaching, repeated application in a season, less efficiency, and less bioavailability to plants [97]. Nanofertilizers efficient enough to overcome these drawbacks can be extremely cost efficient when applied in large-scale field programs.

Similarly, though NPs application increase the grain nutrient content in plants making them a chief source for human health and alleviating human malnutrition, their supply and extent of accumulation should be carefully monitored as their excess intake can lead to potential health risks [98,99]. Nevertheless, a number of studies specified the efficiency of NPs in improving wheat nutritional value, the particular influence developed on human health from the consumption of these fortified parts has not been well explored yet and need to be thoroughly studied [96,98]. Wheat nanobiofortification should be employed in the light of recommended daily allowance of nutrients for human that is 1000 mg Ca, 3000–4000 mg K, 700 mg P, 200 mg, 1500 mg Na, 2.3 mg Mn, 1.2 mg Cu, 1–3 mg Fe, and 36–150 µg Zn [5,100]. The natural properties of different nanomaterials such as their required dosage concentrations, material types, coatings, solubility, size, shape, and surface area can contribute to the severe health risks to human beings [49]. The chemical and physical strategies for NPs synthesis can have greater toxicity as compared to the biogenic synthesis of nanoparticles. NPs have enormous potential to increase wheat grain nutrient content upto the required extent, however, a deep understanding of its functioning would promote its safe usage in wheat biofortification. Due to the metallic nature of most of the NMs, these could be the source of metal toxicity in soil, thus, their dosages, types and application methods should be carefully monitored before application. Additionally, due to their smaller size, NPs can be easily absorbed by plants and enter in food chain increasing the risks of health hazards. Thus, scientists, growers, and consumers should be carefully educated about the do’s and don’ts of nanofertilized wheat crops and NPs should be used for wheat biofortification under the regulatory framework [49].

## 12. Conclusions and Future Perspectives

Though there are reports of toxicity of some of the nanomaterials used in agriculture, they remain one of the best options to provide solutions to major targeted problems in agriculture. Nanotechnology not only improves understanding of different mechanisms in plants but also helps in controlling diseases and regulating abiotic stresses consequently increasing the yields and nutritional value. While having these advantages, the use of NFs in agriculture also facilitates the reduction of environmental pollution with the controlled delivery of nutrients at the appropriate time. Nanotechnology-based methods assist in the evaluation of real-time growth of crops and provide the required information for precision farming such as the time of fertilizer application and sowing [101]. Several studies specified and confirmed the positive role of nanomaterials in alleviating the biotic and abiotic stress-induced changes in wheat crops [102,103,104,105,106]. However, in many directions, still it is required to turn the theoretical properties into applications.

Nanotechnology-based biofortification is one of such areas of wheat development which even after getting positive results in a number of studies could not be thoroughly applied in field-scale programs. However, applying the results obtained from pots and small experiments to the fields could largely facilitate food security, nutritional development, and a sustainable environment simultaneously. Other than the nanomaterials that have been mentioned for wheat biofortification in this review in detail, there are several NPs that are actively being used for handling the biotic and abiotic stresses, but could not be explored at all for wheat biofortification. The intercalated NPs such as nanozeolites and nanoclays, which can restore soil fertility and enhance the efficacy of applied fertilizers, can be an efficient source of wheat biofortification. Similarly, the frequently focused nanomaterials such as TiO_2_, CeO_2_, Ag, Au, platinum, Se, Co, carbon nanotubes, fullerol, fullerene should also be tested and employed for enriching the wheat grains. Moreover, to utilize the most eco-friendly, biocompatible, and non-toxic forms for wheat biofortification, biological nanomaterials based on green nanotechnology or phytonanotechnology can be preferred. Different plant organs such as stems, leaves, roots, barks, and fruits can be utilized for synthesizing biological nanomaterials. Plant compounds including acids, saponins, proteins, carbohydrates, terpenoids, phenolics, and flavonoids can reduce different minerals or mineral oxides to their nanoparticulate structures. Despite the possibilities of using several chemically and biologically synthesized nanomaterials for wheat biofortification, it is extremely essential to assess their biosafety level before employing them in the fields [85]. Due to the wide acceptance of nanotechnology for wheat development, a rigorous evaluation of the safety of the nanomaterials can largely improve its application scale in biofortification programs. Wheat biofortification in agricultural lands using nanomaterials can largely ensure sustainable food availability, a less polluted environment, and improved nutritional health, both in the developed and developing world.

## Figures and Tables

**Table 1:** Summary of the studies conducted using different forms nanomaterials for wheat nano-biofortification and the obtained impact in terms of nutrient content in seed grain in comparison to the control (no nanomaterial applied) and conventional agronomic biofortification applications (wherever present). (NC = No comparison was done with conventional fertilizers in this study).

Reference	Type of Application	Place of Trial	Growth Medium	On Application of Nanomaterial				On Application of Conventional Fertilizers		
				Applied Nanomaterial	Amount Applied	Nutrient Content in Grain without Application of Nanomaterial	Nutrient Content in Grain on Addition of Nanomaterial	Applied Conventional Fertilizer	Amount Applied	Nutrient Content in Grain on Addition of Conventional Fertilizers
Deshpande et al. 2017	Foliar	PVC columns	Sand	Zn-CNP	≈20 mg g^−1^, ≈25mL	≈15–19 µg g^−1^ DW Zn	≈21–25 µg g^−1^ DW Zn	NC		
Xue et al. 2017	Soil	Pot	Soil	Nanochitin	0.006 g kg^−1^	184.3 g kg^−1^ protein 58.6 mg kg^−1^ Fe 42.13 mg kg^−1^ Zn	204.1 g kg^−1^ protein 65.39 mg kg^−1^ Fe 51.45 mg kg^−1^ Zn	NC		
Zhang et al. 2017	Foliar	Field	Soil	ZnO NP	2 g L^−1^ at a rate of 1.2 kg ha^−1^	18.4 mg kg^−1^ (Year 1) Zn 23.6 mg kg^−1^ (Year 2) Zn	26.5 mg kg^−1^ (Year 1) Zn 34.6 mg kg^−1^ (Year 2) Zn	ZnSO_4_	7 g L^−1^ at a rate of 4.2 kg ha^−1^	21.1 mg/kg (Year 1) Zn 29.5 mg kg^−1^ (Year 2) Zn
Dapkekar et al. 2018	Foliar	Field	Soil	Zn-CNP	40 mg L^−1^	39.5 µg g^−1^	53.3 µg g^−1^	ZnSO_4_	400 mg L^−1^	59.40 µg g^−1^ Zn
Munir et al. 2018	Seed Priming	Pot	Soil	ZnO NPs	100 mg L^−1^	≈12 mg kg^−1^ DW Zn	≈20 mg kg^−1^ DW Zn	NC		
Burhan and Al-Hassan et al. 2019	Foliar	Field	Soil	Nano NPK	750:90:600 mg L^−1^	-	13.5 % protein	traditional NPK	400 kg ha^−1^ urea, 200 kg ha^−1^ tri super phosphate, 100 kg ha^−1^ K_2_SO_4_	10.68 % protein
Hussain et al. 2019	Foliar	Pot	Soil	Fe_3_O_4_ NP	20 mg kg^−1^	≈40 mg kg^−1^ DW Fe	≈120 mg kg^−1^ DW Fe	NC		
Hussain et al. 2019	Soil	Pot	Soil	Fe_3_O_4_ NP	20 mg kg^−1^	≈40 mg kg^−1^ DW Fe	≈90 mg kg^−1^ DW Fe	NC		
Khan et al. 2019	Soil	Field	Soil	ZnO NP	100 mg kg^−1^	≈20 mg kg^−1^ DW Zn	≈45 mg kg^−1^ DW Zn	NC		
Rizwan et al. 2019	Seed Priming	Pot	Soil	ZnO NPs	100 mg L^−1^	≈15 mg kg^−1^ DW Zn	≈30 mg kg^−1^ DW Zn	NC		
Rizwan et al. 2019	Seed Priming	Pot	Soil	Fe NPs	20 mg L^−1^	≈15 mg kg^−1^ DW Fe	≈30 mg kg^−1^ DW Fe	NC		
Sundaria et al. 2019	Seed Priming	Pot Greenhouse	Soil	Fe_2_O_3_ NP	600 ppm	≈30–40 mg kg^−1^ DW Fe	≈40–45 mg kg^−1^ DW Fe	NC		
Adrees et al. 2020	Soil	Pot	Soil	Fe_2_O_3_ NP	100 mg kg^−1^	≈20 mg kg^−1^ DW Fe	≈45 mg kg^−1^ DW Fe	NC		
Adrees et al. 2021	Foliar	Pot	Soil	ZnO NP	100 mg L^−1^	≈22 mg kg^−1^ DW Zn	≈45 mg kg^−1^ DW Zn	NC		
Astaneh et al. 2021	Soil	Field	Soil	Nano-chelated nitrogen	240 kg ha^−1^	-	69% protein 80 mg P 38 mg K	Urea	240 kg ha^−1^	17% protein 54 mg P 27 mg K
Hussain et al. 2021	Foliar	Field	Soil	Fe_3_O_4_ NP	5 mg L^−1^	≈30 mg kg^−1^ DW Fe	≈45 mg kg^−1^ DW Fe	NC		
Hussain et al. 2021	Foliar	Field	Soil	ZnO NP	25 mg L^−1^	≈18 mg kg^−1^ DW Zn	≈25 mg kg^−1^ DW Zn	NC		
Sheoran et al. 2021	Foliar	Pot	Soil	ZnO NP	120 ppm	17.48 mg g^−1^ FW Protein	22.71 mg g^−1^ FW Protein	Chemical Zn	-	19.91 mg g^−1^ FW Protein
